# MR-compatibility study of a preclinical digital PET/MRI insert

**DOI:** 10.1186/2197-7364-1-S1-A2

**Published:** 2014-07-29

**Authors:** Jakob Wehner, Björn Weissler, Peter Dueppenbecker, Pierre Gebhardt, Benjamin Goldschmidt, Christoph Lerche, Andre Salomon, David Schug, Volkmar Schulz

**Affiliations:** Department of Physics of Molecular Imaging Systems, Institute for Experimental Molecular Imaging, RWTH Aachen University, Aachen, Germany; King’s College London, London, UK; Philips Research Europe, Aachen, Germany; Philips Research Europe, Eindhoven, The Netherlands

The combination of PET and MRI into a hybrid device is challenging since both systems might interfere with each other. Therefore, the study of the MR-compatibility of such a combined device is crucial to understand and solve potential interference phenomena. Interference problems, which typically occur and are reported by several groups, range from B_0_ distortion over Signal-To-Noise Ratio (SNR) degradation to gradient disturbances. In this work, we present the current system status and investigations on the interference between a fully digital PET insert and a 3T clinical MRI. The Hyperion-II^D^ PET/MR insert is designed to fit into a clinical MRI system and consists of 10 PET modules which are equipped with up to 6 detector stacks whereas one of them employs a 4×4 digital SiPM array for photon detection. For MRI acquisition, the PET insert is equipped with a dedicated PET transparent Tx/Rx mouse RF-coil. To study the influence of the PET insert on the MR performance, SNR measurements using SE sequences as well as dedicated noise scans were performed. The B_0_ field distortion caused by the PET insert was studied with field mapping and spectroscopic methods. The influence on the PET performance due to MR operation was evaluated by acquiring PET data from different phantoms (point sources and structured phantoms) while applying demanding gradient and RF dominated stress tests as well as normal MR imaging sequences (SE, FFE, EPI and TSE sequences). All experiments were performed with a partly (one of three possible rings) and a fully populated version of the Hyperion-II^D^ scanner. The presence of the PET detector causes MR performance degradation on an acceptable level. The same holds true for the PET performance. However, MR stress tests reveal some sensitivity of the PET’s electronic to gradient switching. Details about this study will be presented at the conference.

